# Scoring the number of B chromosomes in *Zea mays* L. using droplet digital PCR assay

**DOI:** 10.1186/s13007-023-01019-9

**Published:** 2023-05-03

**Authors:** Radim Svačina, Lucie Hloušková, Miroslava Karafiátová, Jan Bartoš

**Affiliations:** 1 Institute of Experimental Botany of the Czech Academy of Sciences, Centre of Plant Structural and Functional Genomics, Šlechtitelů 31, 779 00 Olomouc, Czech Republic; 2 Department of Cell Biology and Genetics, Faculty of Science, Palacký University, Šlechtitelů 27, 779 00 Olomouc, Czech Republic; 3 Present Address: Plant Science Program, Biological and Environmental Science and Engineering Division, King Abdullah University of Science and Technology (KAUST), Thuwal, 23955-6900 Saudi Arabia; 4 Present Address: KAUST Center for Desert Agriculture, King Abdullah University of Science and Technology, Thuwal, 23955-6900 Saudi Arabia

**Keywords:** Maize, ddPCR, B chromosome, FISH, Direct PCR

## Abstract

**Background:**

B chromosomes are classified as dispensable genomic components tolerated by cells, which are transmitted to progeny despite providing no benefit in most cases. They have been observed in over 2800 species of plants, animals and fungi, including numerous maize accessions. As maize is one of the most important crops worldwide, research on the maize B chromosome has been pioneering in the field. The characteristic of the B chromosome is its irregular inheritance. This results in offspring with a different number of B chromosomes compared to the parents. However, the exact number of B chromosomes in the studied plants is a crucial piece of information. Currently, assessing the number of B chromosomes in maize largely depends on cytogenetic analyses, which are laborious and time-consuming. We present an alternative approach based on the droplet digital PCR technique (ddPCR), which is faster, more efficient and provides the results within one day with the same level of accuracy.

**Results:**

In this study, we report a rapid and straightforward protocol for determining the number of B chromosomes in maize plants. We developed a droplet digital PCR assay using specific primers and a TaqMan probe for the B-chromosome-linked gene and a single-copy reference gene on maize chromosome 1. The performance of the assay was successfully verified by comparison with the results of cytogenetic analyses performed in parallel.

**Conclusions:**

The protocol significantly improves the efficiency of B chromosome number assessment in maize compared to cytogenetic approaches. The assay has been developed to target conserved genomic regions and can therefore be applied to a wide range of diverged maize accessions. This universal approach can be modified for chromosome number detection in other species, not only for the B chromosome but also for any other chromosome in aneuploid constitution.

## Background

Maize (*Zea mays* L.) is a monocot plant of considerable agronomic importance that has served as a genetic model system for over a century [3]. Its genetic information is organized in 20 chromosomes in a diploid constitution (2n = 2x = 20). However, in some accessions, the karyotype is "enlarged" by the presence of so-called supernumerary B chromosomes [18]. B chromosomes have been observed in nearly three thousand species of plants, animals and fungi (D'Ambrosio et al*.*, [6]). These chromosomes are not essential and to maintain themselves in the population, they exploit a non-Mendelian mode of inheritance—in plants, most commonly through post-meiotic non-disjunction in the gametophyte [14]. These supernumerary chromosomes can have positive, negative, or neutral effects on their host and are often seen as parasitic elements that abuse the cellular machinery to replicate and transmit themselves [4, 15, 19]. In maize, as an example, the presence of B chromosomes has been shown to influence gene expression levels [10, 11, 21]. B chromosomes have been the target of countless studies due to their dispensability, making them an attractive tool for studying their impact on the host plant, acting as a mediator for introducing new traits [13]. Furthermore, the mechanisms contributing to maintaining B chromosomes in a population are partly based on processes contradicting generally accepted biological laws in all living organisms. As such, they represent an exciting target for fundamental research [22].

The studies of B chromosomes of maize also significantly aided our understanding of centromere functions [12] and transmission of univalents during meiosis [5, 8, 7]. In most of these studies, knowing the number of B chromosomes in the analyzed lines is crucial. Still, the non-Mendelian inheritance causes irregularities in their number in the progeny, complicating this step [14]. Therefore, assessing B chromosome numbers in plant material is an integral part of such studies. To date, B chromosome dosage in maize has mostly relied on cytogenetic analyses such as chromosome staining or fluorescent in situ hybridization (FISH). These techniques are laborious, skill-intensive, low-throughput and require actively dividing tissues such as root tips. In this context, droplet digital PCR (ddPCR) offers an elegant solution to simplify the assessment of B chromosomes not only in maize but also in many other species.

Using PCR-based techniques avoids the main bottleneck in cytogenetic workflows, which typically restricts the analysis to specific tissue types limited to particular stages of plant development. PCR-based techniques require only a DNA sample, which promotes the abundance of material suitable for the ddPCR approach in maize, where B chromosome is present in all tissues. In this technique, the DNA molecules isolated are randomly distributed into individual droplets. These droplets contain reagents necessary for PCR amplification with TaqMan probe in reference and target locus. The successful amplification inside a droplet indicates the presence of a template DNA molecule for either reference or the target locus and results in a distinctive fluorescent signal detected by an analyzer. The ratio of droplets positive to the reference and target locus is used for copy number variation calculation considering the Poisson distribution [9, 20]. The protocol exploiting the ddPCR technique offers a routine use delivering rapid results, with 96 samples per analysis available in one experimental run. It benefits from the microfluidic analysis of thousands of droplets in each sample, ensuring the accuracy and sensitivity necessary for daily reproducibility.

## Results and discussion

### Droplet digital PCR optimization for B chromosome number scoring in B73 line

The ddPCR assay consisted of two pairs of primers, one pair specific for an A chromosome located reference locus and one pair specific for the B chromosome-specific target locus, with two TaqMan probes specific for one of the two amplicons, each labeled with either FAM [5(6)-carboxyfluorescein] or VIC (2′-chloro-7′phenyl-1,4-dichloro-6-carboxy-fluorescein). The reference primer pair was designed for a single-copy alcohol dehydrogenase gene Zm00001eb056510 located on maize chromosome 1, which is present in two copies in all diploid cells of somatic tissues. The target primer pair was designed for a single-copy B-specific gene Zm00044a000147 located in the proximal region of the long arm of the B chromosome [2].

**Fig. 1:**
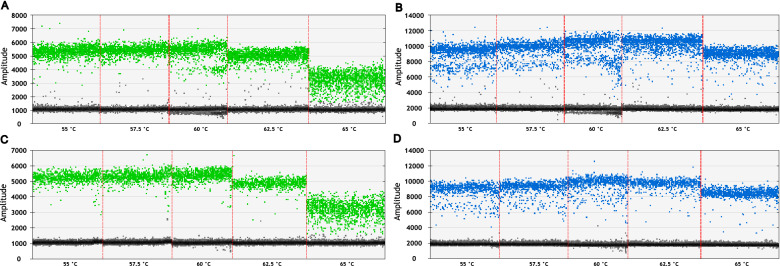
Droplet fluorescence in ddPCR temperature gradient using digested and non-digested DNA. Digestion using *Hae*III and different temperatures of annealing/extension step were tested in the temperature gradient analysis. The fluorescence of each droplet is shown as a dot on the graph, with colored droplets being positive (green for reference; blue for target) and grey being negative. Annealing/extension temperatures are shown below each sample (x-axis), separated by a red line. The undigested DNA showed a less focused signal for both reference (**A**) and target (**B**) probes. The *Hae*III-digested DNA had a better signal-to-noise ratio in both reference (**C**) and target (**D**) channels

The ddPCR conditions were optimized using DNA from the B73 maize line, which contained two B chromosomes. The necessity of DNA digestion by restriction enzyme *Hae*III and temperature of annealing/extension were tested in gradient analysis. A comparison of droplet fluorescence and corresponding background showed the most apparent differentiation between positive and negative droplets at 60 °C for the annealing/extension step of ddPCR in digested DNA (Fig. 1).

### Reliability of the established ddPCR assay

**Fig. 2:**
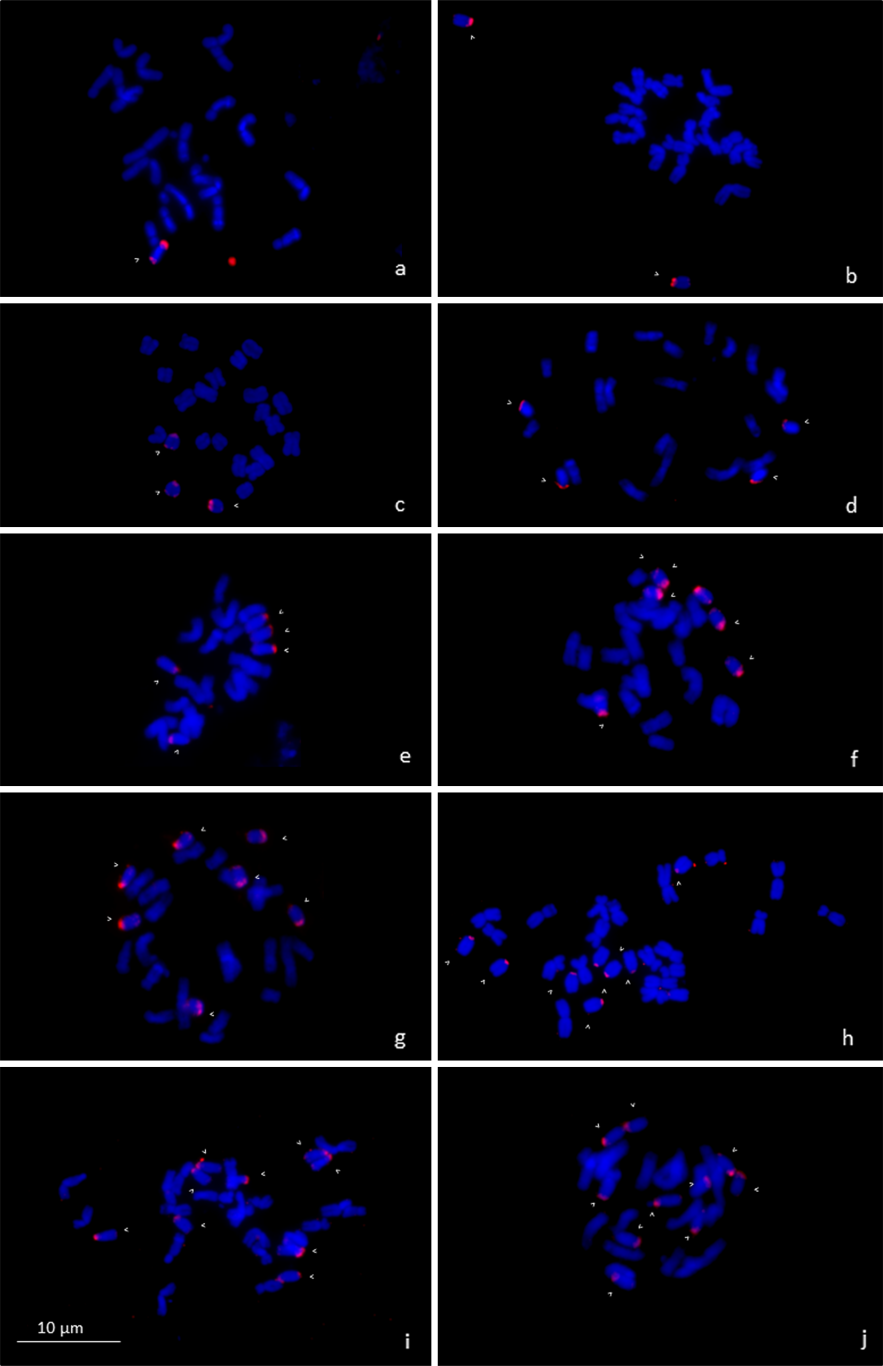
Verification of the number of B chromosomes in the material used for ddPCR optimization. The set of plants of reference line B73 with the respective B chromosome numbers from 1 to 10B (**a**–**j**). B chromosomes are marked with B-specific repeat ZmBs (red; indicated by arrowheads). Chromosomes are counterstained with DAPI (blue)

**Fig. 3:**
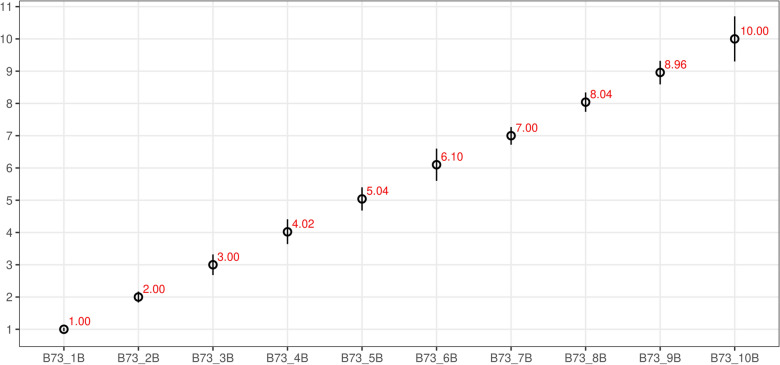
Copy-number results of B chromosome scoring in reference set using ddPCR technique. The value of the estimated number of B chromosomes (copy number; Y-axis) as calculated from ddPCR analysis in each plant (ID in x-axis) over the entire range of values examined. Each estimate is based on a CNV measurement from a single ddPCR well of  > 10,000 droplets. Error bars indicate the Poisson 95% confidence intervals for each copy number measurement

The reference set of plants was selected based on direct PCR pre-selection and cytogenetic screening of root meristems. The exact number of B chromosomes in individual plants was determined by FISH using ZmBs as a B-specific probe [1]. Maize plants with one to ten B chromosomes were selected to test the accuracy of the assay. The B chromosome status of the reference set of plants is summarized in Fig. 2. The identical plants were used to test the performance of the ddPCR assay. The ddPCR reaction was performed with a previously optimized annealing/extension step at 60 °C. The number of B chromosomes calculated from the ddPCR assay was consistent with the number of chromosomes determined by FISH for all samples, with a maximum difference in copy number of 0.1. Notably, the confidence interval of the estimated copy number increased with a higher B chromosome copy number, reaching a maximum for plat with ten B chromosomes (Fig. 3).

### Droplet digital PCR performance in wild maize accessions

**Fig. 4:**
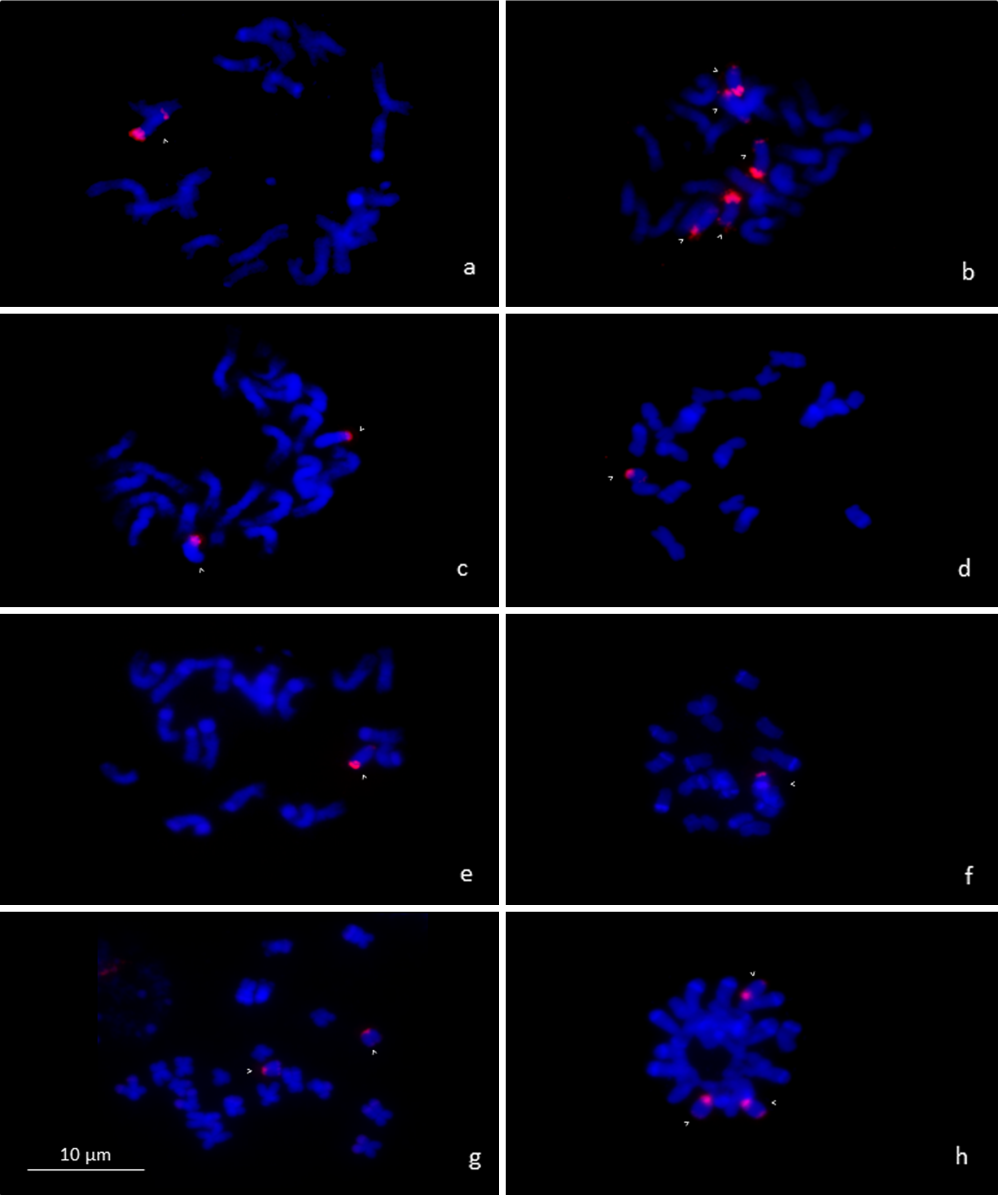
Determination of B chromosome number based on FISH using B-specific repeat ZmBs in the set of wild landraces. **a** ARGE_542 (1B), **b** BOLI_969 (5B), **c** ECUA_693 (2B), **d** GUAT_344 (1B), **e** HOND_52 (1B), **f** RDOM_261 (1B), **g** SALV_70 (2B), **h** YUCA_148 (3B). Chromosomes are counterstained with DAPI (blue). White arrowheads indicate B chromosomes with a B-specific probe (red)

**Fig. 5:**
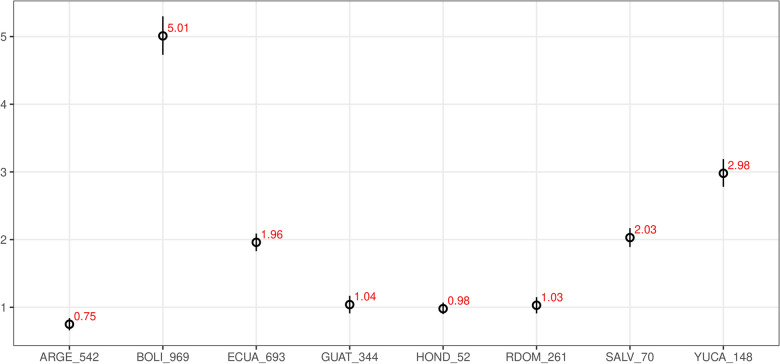
Scoring the B chromosome copy number using ddPCR technique in wild maize landraces. Each estimate of the number of B chromosomes (Y-axis) is based on a CNV measurement from a single ddPCR well of  > 10,000 droplets. Error bars indicate the Poisson 95% confidence intervals for each copy number measurement

To test the robustness of the protocol, the set of seeds from the eight different wild landraces with an unknown number of the B chromosomes were selected for independent ddPCR and FISH scoring of the B chromosomes. Seven of the eight samples showed identical B chromosome numbers determined by FISH and ddPCR, with a maximum difference in estimated copy number of 2.5%, i.e. 0.04 (Table 1, Figs. 4 and 5). However, in a single landrace ARGE_542, the error of the ddPCR estimate was much higher (0.25). The repeated ddPCR analysis in this landrace confirmed the detected deviation. The difference was probably due to a biological rather than a technical reason. Such a deviation may reflect a real ratio of the reference and target genes in this landrace, which could result from a copy number variation of reference/target gene. Alternatively, the number of B chromosomes might not be stable in all cells and the tissues analyzed show some degree of chimerism. Despite this exception, the results obtained from ddPCR confirmed the validity of the presented protocol for B chromosome assessment in different lines and landraces of maize (Table 1).

**Table 1:** Number of B chromosomes in maize landraces estimated by FISH and ddPCR

Accession	CIMMYT ID	# Bs (FISH)	# Bs (ddPCR)	95% confidence interval
ARGE_542	CIMMYTMA 2760	1	0.75	0.66–0.84
BOLI_969	CIMMYTMA 7865	5	5.01	4.73–5.30
ECUA_693	CIMMYTMA 8685	2	1.96	1.83–2.09
GUAT_344	CIMMYTMA 1098	1	1.04	0.91–1.17
HOND_52	CIMMYTMA 876	1	0.98	0.90–1.07
RDOM_261	CIMMYTMA 1321	1	1.03	0.91–1.15
SALV_70	CIMMYTMA 924	2	2.03	1.91–2.17
YUCA_148	CIMMYTMA 2357	3	2.98	2.78–3.19

## Conclusions

In studies focusing on B chromosomes, the number of accessory chromosomes present in individual plants is a fundamental knowledge for subsequent analysis. Unfortunately, due to non-Mendelian inheritance, the number of B chromosomes in the progeny is unpredictable. In this study, we present a novel method for estimating the number of B chromosomes in maize using droplet digital PCR, which significantly improves the efficiency of this analysis. Compared to traditional cytogenetic protocols, our approach speeds up the whole process and does not require actively dividing cells. B-chromosome number assessment using droplet digital PCR can be performed from DNA isolation to results in a single day for a large number of samples. Furthermore, the approach described here is universal and can be adapted for chromosome counting in any species by simply redesigning the ddPCR assay [23].

## Methods

### Plant material

The plant material consisted of *Zea mays* B73 lines with a variable number of B chromosomes, which were used to optimize the conditions and test the reliability of the ddPCR assay. The original seed stock was provided by Prof. James Birchler, University of Missouri, Columbia, MO, USA. The plant material used to test the robustness of the ddPCR assay consisted of wild landraces provided by International Maize and Wheat Improvement Centre (CIMMYT, Mexico) (Table 1). Plants were grown in pots under controlled conditions at 25/18 °C (day/night) with a 16-h photoperiod.

### Direct PCR

The presence/absence of B chromosomes in the individual seedlings was determined before performing other experiments. Phire Plant Direct PCR (Thermo Fisher Scientific, Waltham, USA) was used to select and exclude any maize individuals lacking B chromosomes from the analysis. The presence of B chromosomes was assessed by PCR using three B-specific markers B008, B432 and B452. A small leaf disc (approximately 2 mm^2^) was cut from a seedling leaf and transferred to 20 μl dilution buffer. The sample was incubated at 95 °C for 10 min to obtain a crude DNA extract. The PCR mix contained 1X Phire Plant Direct Master Mix, 1 μM primers (Table 2), 0.5 μl crude DNA extract and ddH_2_O up to 10 μl. The PCR was performed as follows: initial denaturation 98 °C/5 min; 35 cycles of 98 °C/ 5 s; 65 °C/ 5 s; 72 °C/ 20 s, followed by a final extension at 72 °C/ 1 min. The PCR products were separated by agarose gel electrophoresis, stained by ethidium bromide and detected with a UV transilluminator.

**Table 2:** Sequence of primers and probes for direct PCR and ddPCR assay

Oligo ID	Modification and oligo sequence 5′–3′
Oligos used in direct PCR	
B008_F	TAGTTCGTCTCCACACACGC
B008_R	CGAGGAGGTCATCGTCATGG
B432_F	ACATCCTGCTGAGCACAATCA
B432_R	TAGCCTGTTCTGCCCTCTCA
B452_F	TTGCCTTGTGCTGGATAGGG
B452_R	GGGAACTGTAGCAGAGTCGG
Oligos used in ddPCR	
ZmADH1-AF123535_F	GAATGTGTGTTGGGTTTGCAT
ZmADH1-AF123535_R	TACTGTACCTTCTTCGAATCTGCTG
ZmADH1-AF123535_probe	VIC-TGCAGCCTAACCATGCGCAGGGTA-QSY
B008_F	TAGTTCGTCTCCACACACGC
B008_R	CGAGGAGGTCATCGTCATGG
B008_probe	FAM-TCCTCCGCTCGACACATGTCCCTG-QSY

### DNA isolation

DNA was isolated from the plants scored as B-positive by direct PCR. DNA isolation was performed using the NucleoSpin Plant II kit (Macherey–Nagel, Düren, Germany). Initially, 100 mg of maize leaves were lyophilized in Labogene Scanvac Coolsafe (Allerød, Denmark) and homogenized in mixer mill Retsch MM301 (Haan, Germany). Subsequent isolation was performed following the manufacturer’s instructions. DNA quality and quantity were checked using a spectrophotometer Thermo Fisher Scientific NanoDrop One (Waltham, USA) and fluorimeter Thermo Fisher Scientific Qubit 4 (Waltham, USA).

### DNA digestion

Prior to ddPCR, genomic DNA from analysed B-positive plants was digested using the *Hae*III restriction endonuclease enzyme (New England Biolabs, Ipswich, MA, USA). The reaction consisted of 1 U of *Hae*III enzyme per 1 μg of DNA in 1X CutSmart buffer conditions (New England Biolabs, Ipswich, MA, USA). The reaction mix was incubated at 37 °C for 1 h and subsequently diluted to a final concentration of 2 ng/μl for ddPCR application.

### ddPCR assay design

The ddPCR assay consisted of two pairs of primers and two TaqMan probes complementary to each amplicon. The reference and target assays were designed based on the maize genome assembly B73 RefGen_v5 (https://plants.ensembl.org) and the B chromosome assembly [2], respectively. The reference primer pair was designed to target a single-copy Alcohol dehydrogenase gene, Zm00001eb056510, located on maize chromosome 1. The target primer pair was designed for a B-specific gene, Zm00044a000147, located in the proximal region of the long arm of the B chromosome (Table 2, [2].

### Droplet digital PCR

Droplet digital PCR was performed using either DNA digested with restriction endonuclease *Hae*III (NEB, Ipswich, USA) or non-digested DNA (in an optimization experiment). The reaction mix was prepared according to the manufacturer's instructions. It contained 1X ddPCR Supermix for Probes (Bio-Rad, Hercules, USA), 900 nM reference primers, 900 nM target primers, 250 nM reference TaqMan probe (VIC), 250 nM target TaqMan probe (FAM), 10 ng DNA and ddH_2_O up to 20 μl. Droplets were generated using a QX200 Droplet Generator according to the manufacturer's instructions (Bio-Rad). PCR amplification within individual droplets was performed in a C1000 Touch™ thermal cycler (Bio-Rad) under the following conditions: all steps with a ramp rate of 2 °C/s: enzyme activation 95 °C/10 min, 40 cycles of 94 °C/30 s; 60 °C/1 min, followed by enzyme deactivation at 98 °C/10 min. Fluorescent signal detection was performed using the QX200 Droplet Reader and subsequent analysis was done in QuantaSoft v. 1.7.4 software (Bio-Rad) using the copy number variation protocol with the number of reference copies set to two (for diploid genomic constitution).

### Fluorescence in situ hybridization

Mitotic metaphase chromosomes were obtained from the root tip meristem. Roots were collected from 3-day-old seedlings and treated with ice-cold water for 48 h. Roots were fixed in 90% ice-cold acetic acid for 10 min and placed in 70% ethanol until use. Chromosome preparations using the drop technique were performed according to Kato et al*.* [17].

The B chromosome was detected using the B-specific tandem repeat ZmBs as a probe [1] in fluorescence in situ hybridization (FISH). The ZmBs probe was labeled with tetramethylrhodamine-5-dUTP (Roche, Manheim, Germany) by PCR using primers F-AGACCCTAAACCCTGAACCC and R-CTGGTGCTAAGTGTTTGGGG. The reaction mix consisted of 50 ng B-positive maize genomic DNA, 1 × standard Taq reaction buffer, nucleotides (0.2 mM each of dATP, dCTP, dGTP; 0.1 mM each of dTTP and dUTP), 1 μM of each primer, 0.5 U Taq DNA Polymerase (New England Biolabs, Ipswich, MA, USA) and ddH2O up to 25 μl. The probe was labeled under the following conditions: initial denaturation 94 °C/10 min; 34 cycles of 94 °C/30 s; 58° C/30 s; 72° C/60 s, followed by a final extension at 72 °C/10 min. FISH was performed as described by Karafiátová et al. [16]. Signals were observed using a Zeiss Axio Imager Z2 fluorescence microscope (Carl Zeiss, Jena, Germany) equipped with a CCD camera.

## Data Availability

Data sharing is not applicable to this article as no datasets were generated or analysed during the current study.
